# Editorial: We are not WEIRD: Chinese culture and psychology

**DOI:** 10.3389/fpsyg.2024.1384290

**Published:** 2024-03-11

**Authors:** Yung-Jong Shiah

**Affiliations:** ^1^Graduate Institute of Counseling Psychology and Rehabilitation Counseling, National Kaohsiung Normal University, Kaohsiung, Taiwan; ^2^Hui Lan College, National Dong Hwa University, Shoufeng Township, Taiwan

**Keywords:** Chinese culture, Chinese traditions, self-cultivation, Confucianism, Taoism, Buddhism

Professor Kwang-Kuo Hwang, my mentor, and I organized this current Research Topic. It is an extended issue of the previous issue titled “*Eastern Philosophies and Psychology: Toward Psychology of self-cultivation*” (Hwang et al., 2017). However, sadly, he passed away peacefully in his sleep on July 30, 2023. The sudden passing of my mentor is genuinely unacceptable and heartbreaking. He often remarked that the research orientation of “logical positivism” is not the biggest obstacle to developing and publishing indigenous social science (Hwang, 2019). He further emphasized that many Chinese scholars unquestioningly adopted Western social science theories without critical thinking, engaging in research that merely mimics existing academic work and neglects Chinese culture. Furthermore, he pointed out that there needs to be more understanding of the essence of Western science, which is scientific philosophy. The biggest misconception is that Western scientific philosophy is confined to logical positivism only.

By providing a thorough scientific interpretation of Chinese traditions and revolutionizing “WEIRD” psychology and social science (Hwang, 2012; Shiah, 2016, 2021, 2023; Kuo et al., 2022; Xu et al., 2022), we can pave the way for a transformative movement. For the sake of establishing an autonomous academic tradition of social science in transformative Confucian culture, we established the Chinese Indigenous Social Science Association in 2018 in Taiwan to promote this movement, and I am the current president of the association. We encourage our colleagues to construct their theoretical models for conducting empirical research in Chinese societies, which is also the primary reason for establishing the current Research Topic.

We decided to increase the visibility of Chinese culture and psychology by publishing our works in an international journal of high reputation, and *Frontiers in Psychology* became our first choice. We called for papers on *Philosophical and Theoretical Psychology* from the international academic community and obtained a total submission of 87 articles. Eventually, 11 articles were accepted for publication after a strict review procedure by FIP standards.

The two following papers are from the theoretical perspective. Chang Azanlansh constructed the Dialectical Mandala Model of Self-cultivation to provide a universal framework for the multifaceted and systematic analysis of self-cultivation traditions, enabling future research to further develop additional culturally specific ontologies and psychological models in the second step of the strategy. This model can assist researchers in making ontological commitments, understanding self-cultivation more comprehensively, and determining whether they have overlooked any research domains. Chen proposed a dual-mode framework of achievement goals to conceptualize the motivation for academic learning, including two kinds of effort beliefs (obligation-oriented and improvement-oriented belief about effort) students may develop when pursuing academic achievement in societies influenced by Confucian-heritage contexts (CHC).

Li et al. pointed out that, according to the role obligation theory of self-cultivation, learners in CHC tend to perceive academic failure from personal and interpersonal perspectives. The fundamental differences in fear of failure further indicated the inadequacy of the self-worth theory in explaining achievement motivation, where relationalism and role obligations are significant parts of the cultural traditions. Fwu et al. found that CHC's teachers who hold an obligation belief tend to attribute students' failure to a lack of fulfilling duties and provide duty-based feedback, including comforting and advisory feedback based on duty, encouraging students to persevere rather than change direction.

Wong and Cowden provide some strategies for advancing a global psychological science that could enrich the WEIRD-centric landscape of current psychological science. Tang et al. found that independence, intention of residential mobility, and relational mobility positively influenced the preference for cosmopolitan cities. Shu et al. targeted the sense of belonging and homeland construction for refugees and their descendants. They ascribed meanings to resettlement sites and experienced specific emotions within them, thereby fostering a sense of place identity and initiating the process of homeland construction.

Liu et al. found a more robust kinship premium in generosity among Chinese than French students and no significant effect of cultural collectivism. Han proposed a “cultural perception + functional satisfaction and burnout + social media” framework to interpret Chinese youth kinship communication activity. Yik and Chen found an inconsistent result to previous findings that Chinese people did not have a higher tendency to report somatic symptoms of their psychological distress than people with a European ethnic background. Qin reported Longtao He's book investigating the experiences and perceptions of filial care among migrant peasant workers who came home from cities to provide care for elderly parents with advanced cancer.

Those articles provide us with solid confidence that the strategy of constructing culture-inclusive theories and their empirical studies by integrating Western and Eastern philosophies opens a new field of psychology.

Finally, I commemorate Professor Huang's contributions to indigenous Chinese social sciences through this Research Topic.

## Author contributions

Y-JS: Writing—review & editing, Writing—original draft.
